# LTI-03 peptide demonstrates anti-fibrotic activity in *ex vivo* lung slices from patients with IPF

**DOI:** 10.1016/j.isci.2025.113437

**Published:** 2025-08-26

**Authors:** BreAnne MacKenzie, Poornima Mahavadi, Yago Amigo Pinho Jannini-Sa, Brecht Creyns, Ana Lucia Coelho, Milena Espindola, Clemens Ruppert, Brian Windsor, Clemens Aigner, Cory M. Hogaboam, Andreas Guenther

**Affiliations:** 1 Rein Therapeutics, Inc., 12407 N. Mopac Expy, Suite 250 #390, Austin, TX 78758, USA; 2 Department of Internal Medicine, Justus-Liebig University, University of Giessen and Marburg Lung Center (UGMLC), Member of the German Center for Lung Research (DZL), 35392 Giessen, Germany; 3 Cedars Sinai Medical Center, 127 S San Vicente Boulevard AHSP, Los Angeles, CA 90048, USA; 4 Department of Thoracic Surgery, Vienna General Hospital, 1090 Vienna, Austria; 5 Lung Clinic, Evangelisches Krankenhaus Mittelhessen, 35398 Giessen, Germany; 6 European IPF Registry/Biobank, 35392 Giessen, Germany; 7 Institute for Lung Health, 35392 Giessen, Germany; 8 Cardiopulmonary Institute (CPI), 35392 Giessen, Germany

**Keywords:** pharmacology, transcriptomics

## Abstract

Idiopathic pulmonary fibrosis (IPF) features excessive extracellular matrix deposition driven by activated fibroblasts and dysregulated signaling pathways. To assess the anti-fibrotic effects of LTI-03, a caveolin-1 scaffolding domain peptide, *ex vivo* precision-cut lung slices (PCLS) from patients with IPF were evaluated using bulk RNA sequencing, Ingenuity Pathway Analysis, and immunofluorescence. LTI-03 dose-dependently reduced collagen protein levels, suppressed pro-fibrotic cytokines, inhibited pro-fibrotic pathways, and activated protective mechanisms such as PTEN and PPAR signaling. Modulation was comparable to nintedanib but without the induction of apoptosis or necrosis pathways. These results demonstrate LTI-03’s potential therapeutic efficacy in a highly relevant translational disease model and support LTI-03 as a promising next-generation therapeutic to halt IPF progression and improve patient outcomes.

## Highlights

• IPF PCLS explants actively undergo fibrotic processes over 5 days
• LTI-03 attenuates pro-fibrotic pathways without inducing necrosis or apoptosis
• LTI-03 suppresses collagen and pro-inflammatory cytokines in IPF PCLS
• Data support LTI-03 as a clinical candidate for IPF therapy

## Graphical abstract

**Figure:**
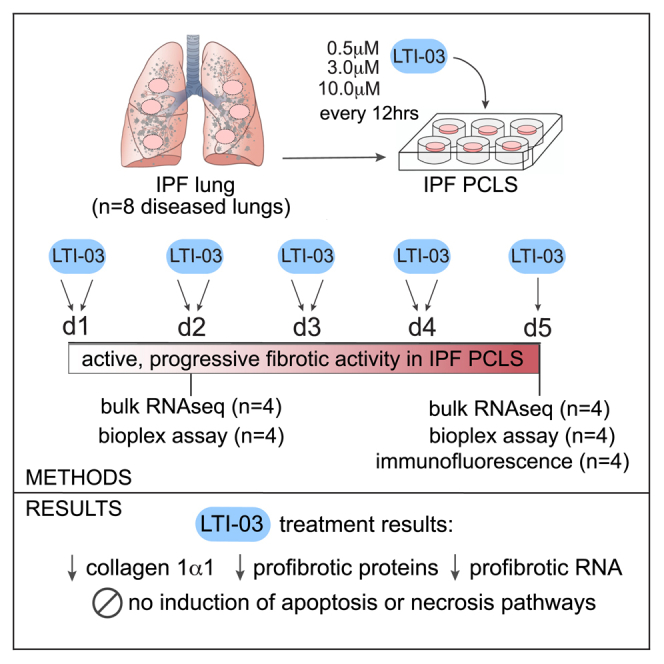

## Introduction

Idiopathic pulmonary fibrosis (IPF) is an interstitial lung disease (ILD) that is estimated to affect over 200,000 people in the United States and 1 out of every 200 adults over the age of 60.^1^ IPF is characterized by progressive interstitial fibrosis of the lung parenchyma, which results in continuous loss of lung function and gas exchange properties. As a result, patients experience dry coughing, progressive dyspnea initially under exercise, later at rest, and, ultimately, death due to respiratory failure. In untreated patients, the median survival from the time of diagnosis is 2–3 years.^1^ Hence, there is a great unmet medical need.

Antifibrotic drug candidates are routinely evaluated in the rodent bleomycin model of lung fibrosis for their ability to reduce extracellular matrix (ECM) protein deposition. However, out of the 700 different drugs tested in the bleomycin model and the 145 clinical trials performed in part based on preclinical results of candidates showing efficacy in this model,^2^ only two drugs, Pirfenidone (Esbriet)^1^^,^^3^ and Nintedanib (OFEV),^4^ are authorized for this condition. While they slow the rate of disease progression and expand survival time, they fail to stop progression or to induce regeneration.

Taken together, embedded in the routinely utilized mouse bleomycin model are striking limitations that impede translation to human clinical disease, including: the use of young, female mice of inbred genetic background, sterile housing, homogenous diet, and spontaneous resolution of bleomycin injury. While these conditions facilitate experimentation and are useful for determining whether a compound confers antifibrotic activity in an *in vivo* model system, the large number of IPF clinical failures suggests that more rigorous translational models are desperately needed to evaluate potential candidates.

Caveolin-1 (CAV1) is an integral membrane protein expressed primarily in epithelial, endothelial, immune cells, and stromal cells of the lung.^5^^,^^6^ CAV1 is required for the formation of plasma membrane invaginations (caveolae)^7^ and is implicated in receptor endocytosis, signaling cascades, membrane transport, and immunity. The loss of CAV-1 mRNA and protein expression has been observed in many cell types of human IPF lung tissue^6^ as well as in many other human tissues affected by fibrotic disease.^8^ In addition, CAV1 expression is reduced in a rodent bleomycin model of pulmonary fibrosis^9^ and *Cav1*^−/−^ mice develop spontaneous pulmonary fibrosis (PF).^10^ A hydrophobic, seven amino acid sequence (FTTFTVT), referred to as CSP7 or LTI-03, has been derived from the broad interacting caveolin scaffolding domain (CSD), which putatively binds to any endogenous proteins predicted to have caveolin binding domains (CBD).^11^ CBDs play a role in cav-1 dimerization as well as the regulation of diverse signaling intermediates, many of which are implicated in the pathogenesis of fibrosis.^6^ LTI-03 was designed to replenish the modulating, homeostatic effects of the CSD domain in the IPF lung. Indeed, LTI-03 was identified based on the inhibitory effect of CSD peptide truncations, deletions, and substitutions on ECM production by human IPF and bleomycin-injured mouse lung fibroblasts and retained the anti-fibrotic activity of full length CSD.^12^ Moreover, in rodent cardiac, pulmonary, and dermal.^13^^,^^14^^,^^15^ fibrosis models, systemic or local administration of LTI-03 prevented or attenuated organ fibrosis. LTI-03 was formulated as an excipient-free dry powder for inhalation for the treatment of IPF.^15^ It mitigated pulmonary fibrosis (PF) in both chronic and acute mouse models^12^ and has demonstrated anti-fibrotic effect in IPF epithelial and fibroblast monocultures^16^^,^^17^ but its activity had yet not been assessed in intact human IPF lung tissue.

In this report, we used human *ex vivo* IPF precision cut lung slices (PCLS) to evaluate anti-fibrotic activity and to analyze the downstream signaling machinery of LTI-03 in an innovative 3D culture of viable IPF lung tissue. Progressive fibrotic activity was observed in untreated human IPF PCLS over 5 days of culture. In IPF PCLS treated with either LTI-03 or the standard of care Nintedanib, we could observe comparable anti-fibrotic activity of LTI-03, as indicated by diminished collagen1a1 immunofluorescence, and reduced proinflammatory and profibrotic transcript and protein expression. LTI-03 had similar pleiotropic antifibrotic effects compared to the standard of care (SOC) treatment, Nintedanib, without any evidence of cellular necrosis or toxicity. We hypothesize that the evaluation of anti-fibrotic activity of potential therapies for IPF in the human *ex vivo* IPF PCLS model may be an important step in predicting a drug candidates’ clinical efficacy.

## Results

### *In silico* identification of putative LTI-03 targets in idiopathic pulmonary fibrosis

LTI-03 is a seven amino acid peptide derived from the CSD region of caveolin-1, which putatively interacts with proteins containing complimentary CBD.^11^ To elucidate the proteins LTI-03 may be interacting with, CBD sites were queried using the MOTIF Search tool (setting: nr-aa) available on GenomeNet (genome.jp).^18^^,^^19^ A total of 7,634 proteins in the human proteome were predicted to contain these CBD sequences (Table S1). Multiple CBD domains were found in the following proteins of relevance to the pathogenesis of IPF: fibroblast growth factors (FGFs), fibroblast growth factor receptors 1–4 (FGFR1-4), frizzled (FZD; WNT receptors), platelet-derived growth factor receptors A and B (PDGFR), and vascular growth factor receptors 1 and 3 (VEGF). FGFR1-4, PDGFA/B, VEGFR1/3 are known targets of Nintedanib^20^ and contain CBDs as shown in Table 1.

**Table 1:** CBD binding motifs relevant for IPF and indication of known targets of Nintedanib

GENE	UNIPROT	CBD motif 1	CBD motif 2	Mixed domain	Nintenadib
		ϕXϕXXXXϕ	ϕXXXXϕXXϕ	ϕXϕXXXXϕXXϕ	Targets
FGF2	P09038	0	1	0	–
FGF5	P12034	0	1	0	–
FGF8	P55075	0	1	0	–
FGF9	P31371	0	1	0	–
FGF11	Q92914	0	1	0	–
FGF12	P61328	1	1	0	–
FGF13	Q92913	1	1	0	–
FGF14	Q92915	1	1	0	–
FGF16	O43320	0	1	0	–
FGF20	Q9NP95	0	4	0	–
FGFR1	P11362	2	1	1	•
FGFR2	P21802	1	1	1	•
FGFR3	P22607	1	1	1	•
FGFR4	P22455	1	1	1	•
FZD1	Q9UP38	1	0	0	–
FZD2	Q14332	1	0	0	–
FZD4	Q9ULV1	1	2	1	–
FZD5	O75084	1	0	0	–
FZD6	Q9H461	3	0	0	–
FZD7	O00144	1	2	1	–
FZD10	Q9ULW2	0	1	0	–
PGFRA	P16234	1	3	1	•
PGFRB	P09619	1	2	1	•
VGFR1	P17948	3	1	1	•
VGFR3	P35916	2	2	1	•

### Profibrotic proteins and transcripts increased in a temporal manner in idiopathic pulmonary fibrosis precision cut lung slice cultures

**Figure 1:**
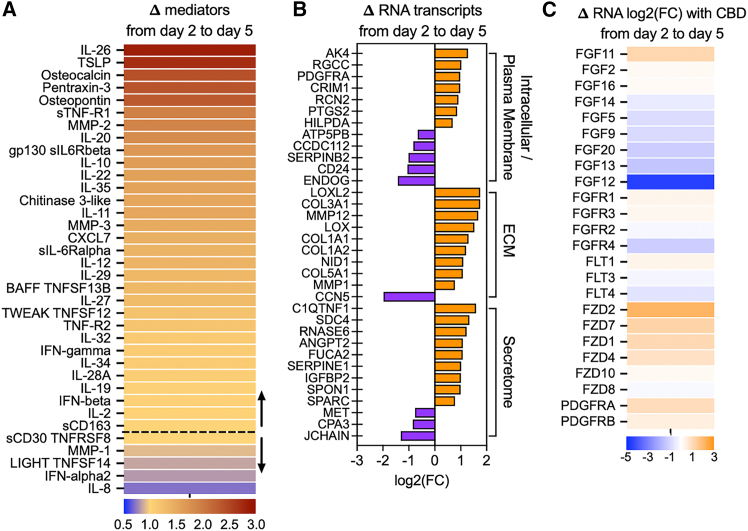
Progressive fibrotic activity was observed in IPF PCLS cultures over 5 days (A) Ratio of the change in the expression of proinflammatory and profibrotic mediators from day 2 to day 5 in supernatant harvested from untreated, IPF PCLS explants (*n* = 8 unique biological samples). Dashed line indicates no change in baseline mediators from day 2 to day 5. Corresponding arrow pointing up indicates mediators increasing in expression over time; arrow pointing down denotes mediators decreasing in expression from day 2 to day 5. (B) Log2 fold change (FC) of gene expression changes related to ECM deposition, secreted factors, and intracellular and plasma membrane factors were increased from day 2 to day 5 according to bulk RNA sequencing of IPF PCLS explants (*n* = 4 unique biological samples). (C) Profibrotic factors containing CBD domains were also increased. No statistics were performed.

To determine whether IPF PCLS exhibited dynamic changes in profibrotic and proinflammatory mediators over the course of the culture time examined in this study, a Bio-Plex Human Inflammation Panel I was used to measure mediators in supernatants at days 2 and 5 of PCLS culture. Most inflammatory and pro-fibrotic mediators were increased on protein level on day 5 compared to day 2, suggestive of a progressive profibrotic activity in PCLS cultures over this time frame (Figure 1A). In addition, transcript analysis via bulk RNA-sequencing revealed that factors relevant to ECM deposition, secreted factors, and intracellular and plasma membrane factors related to fibrosis, were also increased over the time of PCLS culture (Figure 1B). Profibrotic factors containing CBD domains were also increased (Figure 1C). These data provide evidence that the PCLS system adequately reflects the progressive nature of the human disease in the explanted PCLS in the culture dish.

### LTI-03 dose dependent reduction of collagen 1α1 protein in idiopathic pulmonary fibrosis precision cut lung slice tissue following 5 days in culture

**Figure 2:**
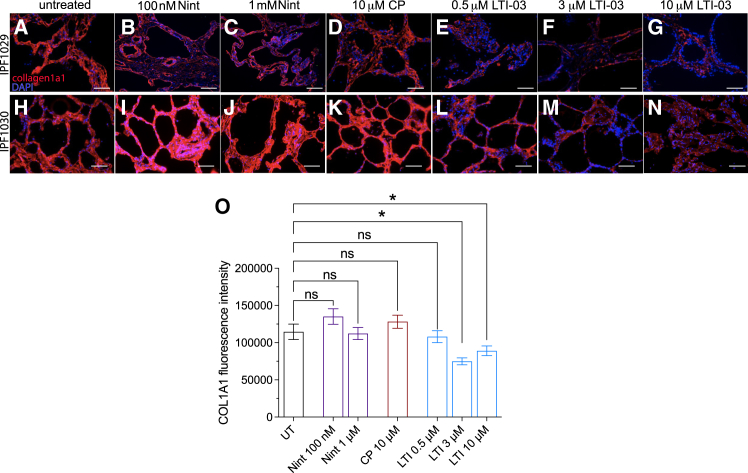
Dose dependent decrease of Collagen1α1 protein in LTI-03 treated IPF PCLS after 5 days in culture (A–O) Representative immunostaining staining of 2 IPF PCLS for collagen1α1 (red) with nuclear DAPI counterstain (blue) and all treatment groups: (A, H); untreated, (B, I); 0.1 μM Nintedanib; (C, J); 1.0 μM Nintedanib, (D, K); control peptide; (E, L); 0.5 μM LTI-03; (F, M); 3.0 μM LTI-03; 10 μM LTI-03; Scale bars 400 μm. (O) Quantification of fluorescence intensity. ∗*p* < 0.01 via Tukey’s multiple test. Error bars represent standard error of the mean.

Diseased, fibrotic lung fibroblasts present in the IPF lung deposit collagen 1α1 (col1α1) extracellular matrix protein which destroys lung architecture and leads to a decline in lung function^21^ (3) In IPF PCLS (*n* = 4), CP (at 10 μM), LTI-03 (at doses of 0.5, 3, or 10 μM) or Nintedanib (0.1 μM or 1 μM) were administered to IPF PCLS every 12 h for 5 days and representative images from two IPF patient PCLS studies (IPF1029 and IPF1030) are shown in Figures 2A–2N. At doses of 3 (Figures 2F and 2M) or 10 μM (Figures 2G and 2N), LTI-03 treatments visibly decreased col1α1 staining intensity compared to the untreated (Figures 2A and 2H) and CP-treated (Figures 2D and 2K) groups. Staining intensity for col1α1 also appeared to be decreased in the 0.1 μM (Figures 2B and 2I) and 1 μM (Figures 2C and 2J) Nintedanib treatment groups. Calculations of fluorescence intensity were performed on a minimum of 5 areas per PCLS section and 3 PCLS technical replicates per treatment group, and these data are summarized in Figure 2O. Both, the 3 and 10 μM LTI-03 treatments significantly decreased col1α1 staining intensity compared with the untreated groups and with CP (Figure 2O).

### LTI-03 inhibits profibrotic and inflammatory mediators in idiopathic pulmonary fibrosis precision cut lung slice supernatants after 2 and 5 days of treatment

**Figure 3:**
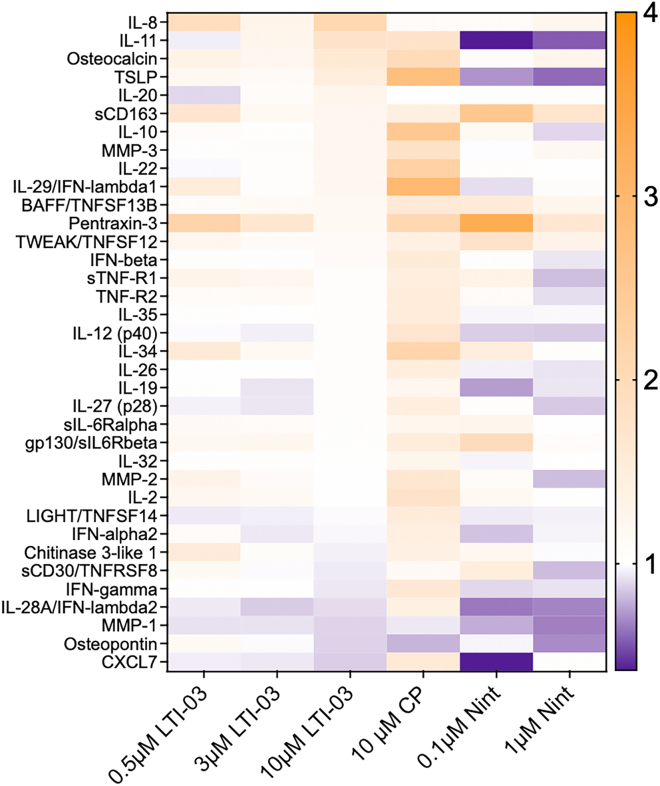
LTI-03 inhibits profibrotic and inflammatory mediators in IPF PCLS following 5 days of treatment Supernatant was harvested from days 4 or 5 post treatment with 0.5 μM, 3.0 μM, 10.0 μM LTI-03, 10 μM CP or 0.1 μM or 1 μM Nintedanib (*n* = 8/PCLS per treatment group, except 0.1 μM Nintedanib; *n* = 4 PCLS). Heatmap indicates the mean values of per protein measured normalized to the mean of untreated controls. No statistics were performed.

Cell free supernatant was removed from PCLS tissue cultures post treatment and analyzed for inflammatory and profibrotic mediators by a human Bioplex panel (Figure 3). The heatmap indicates the mean values per protein measured normalized to the mean of untreated controls. Nintedanib had the strongest, dose-dependent suppressive effect on almost all inflammatory and profibrotic mediators after 5 days and this was followed by LTI-03 at a dose of 10 μM. In contrast, the CP treatment appeared to increase levels of many of these factors. Similar results with all the treatments and controls were observed following day 2 measurements (Figure S1).

### Canonical pathways modulated in idiopathic pulmonary fibrosis precision cut lung slice cultures following nintedanib or LTI-03 treatments

**Figure 4:**
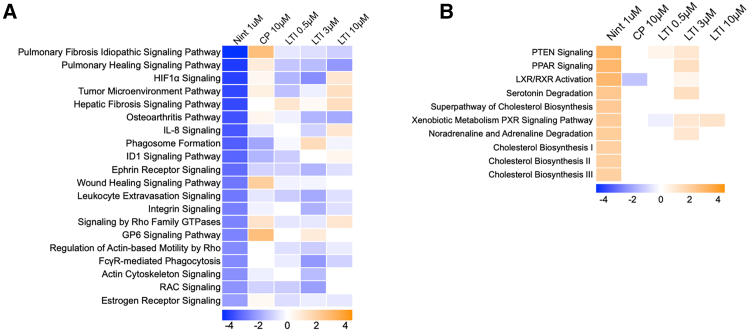
Canonical pathways modulated by Nintedanib or LTI-03 treatment of cultured PCLS (A) Treatment inhibited canonical pathways related to fibrosis signaling in IPF PCLS tissue followed by 5 days of Nintedanib (1.0 μM), control peptide (10.0 μM) or LTI-03 (0.5 μM, 3.0 μM, 10.0 μM) treatment every 12 h (*n* = 4/group). (B) Treatment activated canonical pathways related to fibrosis signaling in IPF PCLS tissue followed by 5 days of Nintedanib (1.0 μM), control peptide (10.0 μM) or LTI-03 (0.5 μM, 3.0 μM, 10.0 μM) treatment every 12 h (*n* = 4/group). All treatments were compared to its respective untreated group and the top inactive or active canonical pathways (*Z* score >2 or < −2 and log_10_p-val >1.3) observed in Nintedanib was used as a comparison for the LTI treatments.

Ingenuity pathway analyses (IPA) of RNAseq datasets revealed that various canonical signaling pathways were modulated in IPF PCLS tissue after 5 days of Nintedanib (0.1 or 1.0 μM), control peptide (10.0 μM), or LTI-03 (0.5, 3, or 10 μM) treatments every 12 h (*n* = 4/group; Figure 4). Treatment suppressed IPA pathways are summarized in Figure 4A, and treatment activated IPA pathways in Figure 4B. All comparisons were calculated between treated and untreated groups. LTI-03 dose-dependently inhibited the Idiopathic Pulmonary Fibrosis Signaling Pathway, which is comprised of transcripts such as TGFB1, VEGFA, PDGFB, EGF, IL6, IL1B, and FGF2 (Figure 4A). Other key fibrosis pathways that were consistently inhibited by LTI-03 included Pulmonary Healing Signaling Pathway, Osteoarthritis Pathway, Ephrin Receptor Signaling, Leukocyte Extravasation Signaling, FcγR-mediated Phagocytosis, and Regulation of Actin-based Motility by Rho (Figure 4A). LTI-03 also upregulated canonical pathways particularly PTEN, PPAR, and Xenobiotic Metabolism PXR signaling pathways, albeit these effects were mostly apparent at the 3 μM dose. Compared with LTI-03 treatments, Nintedanib at a dose of 1 μM exhibited stronger suppression or activation of the canonical pathways summarized in Figure 4. Day 2 analysis summarizing these canonical pathways is available in Figure S2.

## Expression of upstream fibrosis signaling regulators in cultured idiopathic pulmonary fibrosis precision cut lung slice are modulated by nintedanib or LTI-03 treatments

**Figure 5:**
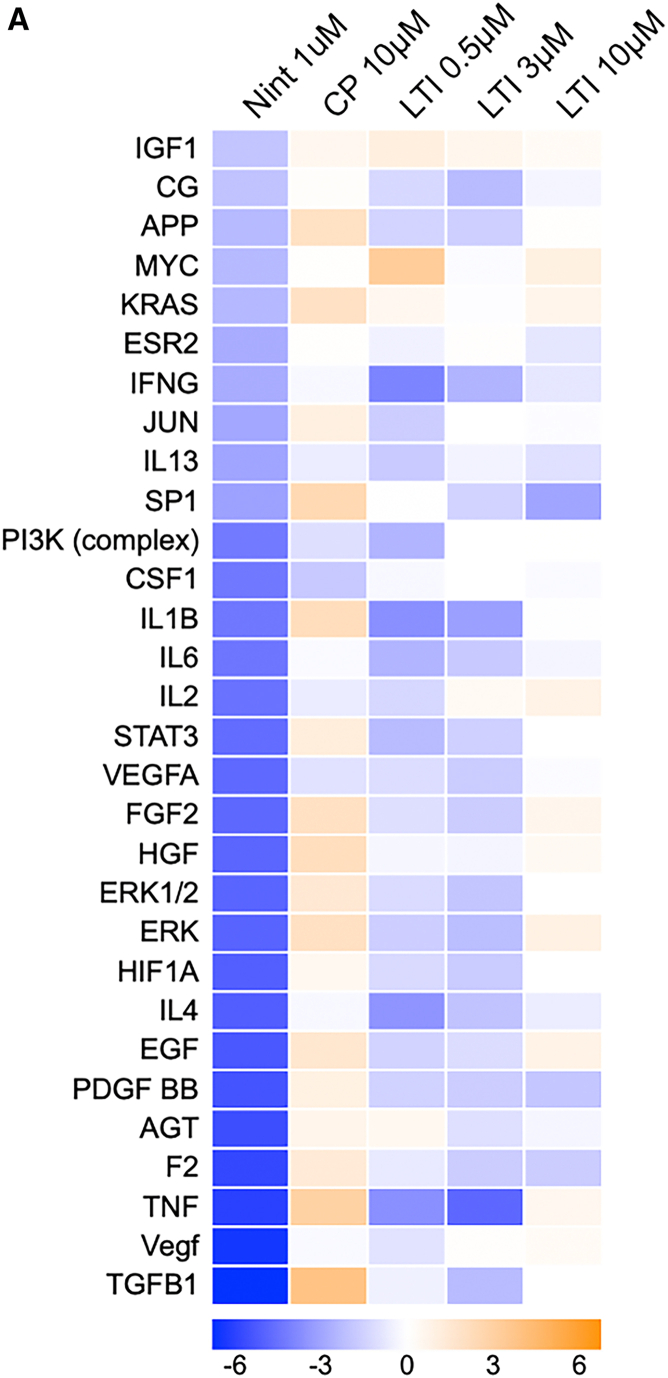
Prediction of inactive upstream regulators in IPF PCLS treated with control peptide, Nintedanib or LTI-03 (A) Prediction of upstream regulators genes related to fibrosis pathway in IPF PCLS tissue followed by 5 days of Nintedanib (1.0 μM), control peptide (10.0 μM) or LTI-03 (0.5 μM, 3.0 μM, 10.0 μM) treatment every 12 h (*n* = 4/group). All treatments were compared to its respective untreated group and the top inactive or active upstream regulators (*Z* score >2 or < −2 and log10p-val >1.3) observed in nintedanib was used as comparison for the LTI treatments.

IPA of RNAseq datasets identified key upstream transcriptional regulators related to fibrosis in IPF PCLS cultures after 5 days of Nintedanib (1.0 μM), control peptide (10.0 μM) or LTI-03 (0.5 μM, 3.0 μM, 10.0 μM) treatment (*n* = 4/group; Figure 5). All the upstream regulators were inhibited by Nintedanib or LTI-03 treatments with the exceptions of insulin growth factor-1 (IGF1), MYC, and KRAS in the LTI-03 treatment groups. Similar to Figure 4, treatment effects of 10 μM LTI-03 appeared to be slightly less pronounced as compared to 3 μM. All values were normalized to untreated groups. Day 2 analysis is available in Figure S3.

### Modulated the expression of disease pathways expression related to fibrotic signaling in idiopathic pulmonary fibrosis precision cut lung slice cultures treated with nintedanib or LTI-03

**Figure 6:**
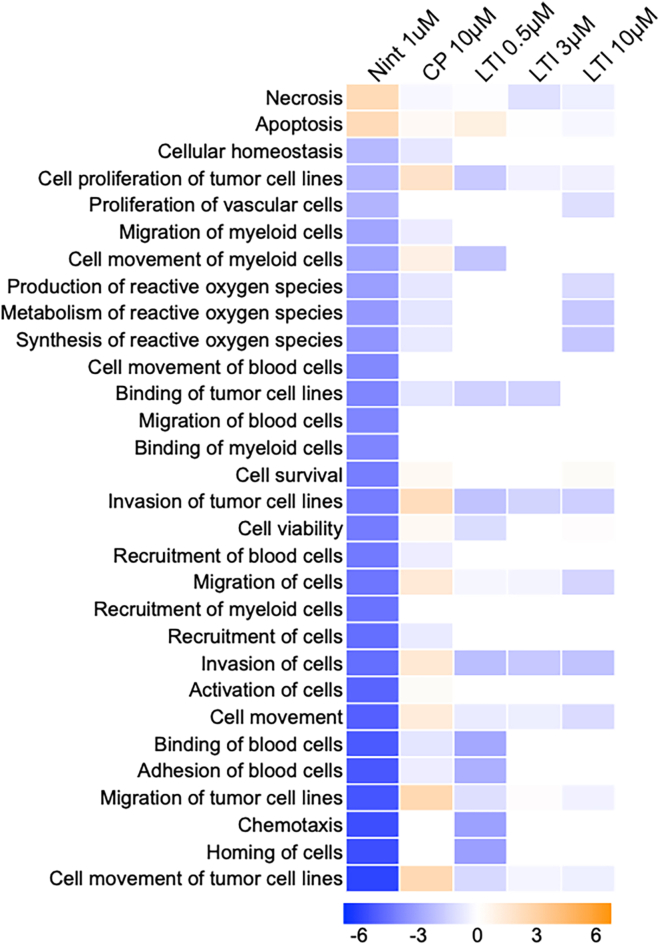
Disease and Functions prediction analysis in IPF PCLS treated with control peptide, Nintedanib or LTI-03 Disease pathway expression related to fibrotic signaling in IPF PCLS tissue followed by 5 days of Nintedanib (1.0 μM), control peptide (10.0 μM) or LTI-03 (0.5 μM, 3.0 μM, 10.0 μM) treatment every 12 h (*n* = 4/group). All treatments were compared to its respective untreated group and the top inactive or active disease and function score (*Z* score >2 or < −2 and log10p-val >1.3) observed in nintedanib was used as comparison for the LTI treatments.

IPA of RNAseq datasets revealed that disease pathway scores were altered in IPF PCLS cultures after Nintedanib (1.0 μM), control peptide (10.0 μM), or LTI-03 (0.5 μM, 3.0 μM, 10.0 μM) treatment for 5 days (*n* = 4/group; Figure 6). Although there were again some discrepancies between the 3 and the 10 μM dose of LTI-03, it appeared as if Nintedanib and CP (to some extent also 3 μM LTI-03) rather increased necrosis and apoptosis pathways, whereas 0.5 and 10 μM LTI-03 suppressed these. Vice versa, cellular homeostasis pathways were rather found to be increased or unchanged in 0.5 or 10 μM LTI-03, but suppressed in Nintedanib and 3 μM μM LTI-03 treated PCLS (Figure 6). In addition, Nintedanib and LTI-03 at 3 μM both decreased transcript expression associated with the Cell proliferation of tumor cell lines, Invasion of tumor cell lines, Invasion of cells, and Cell Movement. Production, Metabolism, & Synthesis of Reactive Oxygen Species seemed to be only downregulated by Nintedanib treatment. All values were normalized to the untreated groups. Day 2 analysis is available in Figure S4.

## Discussion

While countless anti-fibrotic agents are efficacious in animal models of lung fibrosis, most therapies continue to fail in IPF clinical trials.^22^ While many IPF therapies currently in development are designed around single targets,^6^^,^^23^ to date, the two IPF standard of care drugs that have successfully slowed the rate of lung function decline in patients with IPF, target multiple pathways.^24^^,^^25^ While a single target approach may be efficacious in the mouse bleomycin model, they have yet to succeed clinically. Consequently, if translational models should ever become reliable tools for predicting clinical success, they must replicate clinical reality more closely. As such, PCLS generated from explanted IPF patient lungs has emerged as a promising *ex vivo* translational approach to study drug effects directly on IPF tissue.^26^ Embedded in the IPF PCLS model is a state of active disease, progressive fibrosis, the extracellular matrix environment, and a wide range of cell types unique to the IPF lung,^27^ a complex genetic and epigenetic background, and finally, the ability to generate technical replicates from a single tissue core permits the generation of internal control groups. As a result, the interpretation of pharmacological data gathered from the IPF PCLS system has the potential to be more clinically informative and relevant^19^^,^^28^^,^^29^^,^^30^ than other rodent models or *in vitro* monoculture systems.

This IPF PCLS model used in this study was characterized by dynamic temporal changes in both pro-fibrotic proteins and transcripts over five days, reflecting the progressive nature of the disease. Many of the highly upregulated soluble protein factors in IPF PCLS cultures have been described in IPF, including TSLP,^31^ osteopontin, and CXCL7.^32^ Osteopontin is known to bind CD44 and trigger the phosphorylation of FAK, a profibrotic pathway in the PCLS assay.^33^ Transcriptional analysis via bulk RNA sequencing of IPF PCLS also showed that profibrotic intracellular/plasma membrane, ECM, and secreted factors were upregulated in a temporal manner in the IPF PCLS system, again reflecting the perpetuation of fibrosis. Many of the dynamic changes in transcript expression were tied to ECM components, most notably collagens. Thus, the PCLS culture system provides a clinically relevant approach to study pro-fibrotic mechanisms and targeting strategies directed at these mechanisms in IPF.

Due to epigenetic silencing,^9^ loss of transcriptional regulators such as FOXO3A,^23^ and other mechanisms,^8^ caveolin-1 expression is markedly reduced in IPF^6^^,^^9^ and CAV1 is a prognostic predictor in IPF.^34^ While a gene transfer technique has been used to restore cav1 protein expression and ameliorate fibrosis in an experimental lung fibrosis model,^6^ the antifibrotic effects of CSD peptides has been demonstrated in pulmonary fibrosis models^12^^,^^14^^,^^35^ as well as fibrosis due to aging in mice.^36^ CSD peptides have modulatory effects on monocultures of primary IPF fibroblasts^12^ and epithelial cells (unpublished), but the effect of these peptides in primary multi-cellular systems has not been explored to date. To gain insight into the mechanism of action and putative clinical efficacy of LTI-03, we examined its anti-fibrotic activity as compared to the standard of care therapy, Nintedanib. We observed that LTI-03 treatment dose-dependently reduced the deposition of collagen 1α1 protein in IPF PCLS at day 5. While Nintedanib has been shown to reduce COL1A1 transcript expression in normal lung PCLS exposed to a fibrotic cocktail^37^ and modulates neoepitope biomarkers of type III collagen turnover,^38^ we did not observe a statistically significant change in col1α1 protein in the IPF PCLS system after 5 days of treatment, suggesting that IPF PCLS may be a more rigorous experimental model.

**Figure 7:**
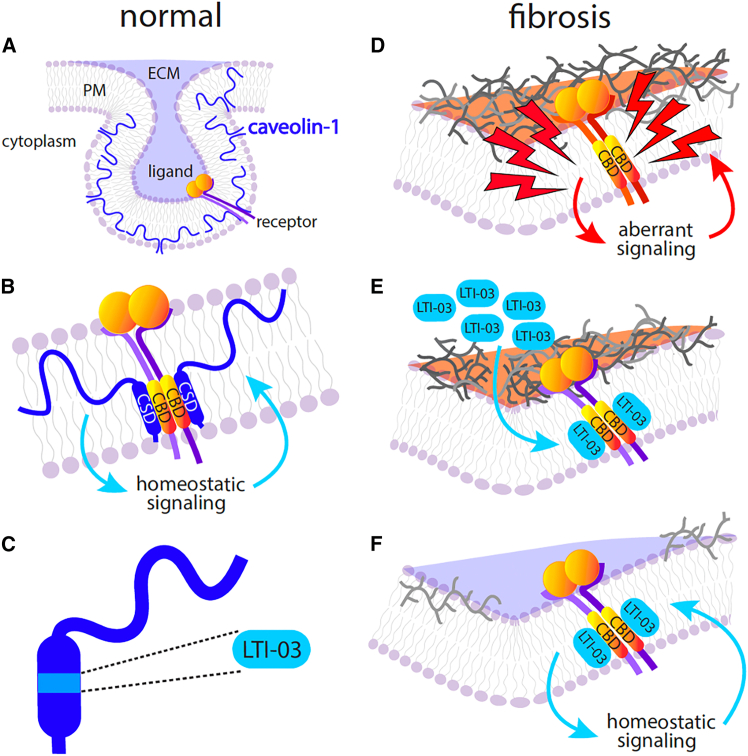
Putative mechanism of LTI-03 anti-fibrotic activity in end stage IPF PCLS (A) In normal lung cells, caveolin-1 (CAV1) is robustly expressed in AEC2s, AEC1s, broncho-epithelium and lung endothelium Caveolin-1 is positioned in the plasma membrane (PM) where it supports the formation of PM invaginations known as caveolae that are thought to be important for mechanical stretching of the lung and which function as receptor sinks. (B) The caveolin scaffolding domain (CSD) is comprised of amino acid sequences 82–101 of the NH_2_ terminal region and plays a role in cav-1 dimerization as well as the regulation of diverse signaling intermediates, many of which are implicated in the pathogenesis of fibrosis. The CSD domain is known to interact with receptors and proteins that contain a caveolin binding domain (CBD) and interaction can result in stabilization, internalization, or recycling of the membrane proteins thereby contributing to homeostatic signaling. (C) LTI-03, the 7-mer peptide used in this study and currently in clinical trials for IPF, is derived from the CSD portion of caveolin-1 (amino acid sequences 89–95). (D) After the initiation of progressive lung disease (indicated by lightning bolts), extracellular matrix proteins are deposited, which perturb lung architecture and generate aberrant signaling and gene expression. Caveolin protein expression in fibrotic lungs is significantly decreased, which likely contributes to aberrant cell signaling. (E) In the context of lung fibrosis, LTI-03 may act as a surrogate CSD, which results in the abrogation of aberrant signaling of local receptors regardless of cell type. (F) The putative interaction between LTI-03 and aberrantly profibrotic receptors or signaling proteins containing CBDs leads to a reduction of profibrotic gene expression and mediators, which results in a decrease in extracellular matrix deposition and return to cellular homeostasis.

LTI-03 has the potential to modulate several canonical intracellular signaling pathways due to its putative interaction with approximately 30% of all proteins; those that contain CBDs.^11^^,^^39^ The seven amino acid sequence of LTI-03 is naturally occurring as it is derived from the CSD portion of cav-1, which is comprised of amino acid sequences 82–101 of the NH_2_ terminal region. This region plays a role in cav-1 dimerization as well as the regulation of diverse signaling intermediates, many of which are implicated in the pathogenesis of fibrosis.^6^^,^^14^^,^^40^ In addition, caveolae work to regulate endocytosis as well as many receptor signaling pathways. In healthy lung cells, endogenous caveolin-1 modulates receptor signaling toward homeostasis via the facilitation of receptor stabilization, turnover and signaling.^5^ Homeostatic modulation is conferred via interactions between CSD of caveolin-1 and the CBD of many receptors and proteins in the plasma membrane. During fibrosis, increased growth factor signaling disrupts cellular homeostasis, leading to the loss of endogenous cav-1 expression and destruction of caveolae. Consequently, we hypothesize that the loss of cav-1 as a homeostatic signaling mediator in the IPF lung is a major contributor to aberrant signaling present in patients with IPF. Our *in-silico* analysis of endogenous proteins containing CBDs highlighted many growth factor receptors, including FGFR2, VEGF, PDFRA/B, among others, which are relevant to IPF pathogenesis.^21^ LTI-03 likely confers the broad attenuation of profibrotic signaling, in part, via its regulatory effects on aberrantly activated growth factor receptors (Figure 7). While direct binding interactions of LTI-03 with these endogenous factors have not been studied to date, herein we provide both protein and transcript analyses suggesting that LTI-03 is targeting multiple receptor-directed pathways in IPF PCLS cultures. Also, the overexpression of caveolin-1 had been shown before to improve barrier function and reduce TSLP expression in cultured human airway epithelial cells.^41^

Stegmayr and colleagues have highlighted the challenges associated with obtaining high yield and high quality RNA for RNA-sequencing of human PCLS.^42^ Like this group, we were able to isolate RNA from the untreated and treated PCLS generated from 4 patients with IPF suitable for RNA-sequencing and IPA analysis in the present study. Nintedanib was more potent than LTI-03 in the modulation of various fibrosis-related canonical pathways and upstream regulators. However, LTI-03 exhibited a dose-dependent breadth of modulation that overlapped considerably with Nintedanib. Notably, in contrast to Nintedanib, LTI-03 did not promote the expression of genes involved in cellular necrosis or apoptosis. Thus, results from the present study support the utility of the IPF PCLS system for RNA sequencing and analysis to address the modulation of fibrotic processes and potential efficacy of new therapeutic approaches for IPF.

### Limitations of the study

While experimental attempts to address direct binding interactions of LTI-03 have been inconclusive, the in-silico analysis revealed thousands of proteins with caveolin-1 binding domains (CBD) to which CSD peptides may putatively bind, suggests that LTI-03’s ability to modulate multiple pathways may be due its ability to interact with many potential targets. Previous work from our group demonstrated that the CSD peptide LTI-2355 (which contains the LTI-03 7-mer sequence) interacts with MRC1, a protein that contains CBD domains, however, additional studies elucidating direct interactions of LTI-03 would further our understanding of its mechanism of action in IPF.^43^ Furthermore, our use of bulk RNA sequencing rather than single cell RNA sequencing prevented us from commenting on any cell-specific transcriptional changes following either treatment.

According to the IPA analysis, canonical pathways were more impacted by 3 μM of LTI-03 rather than by 0.5 or 10 μM. We believe this is in part explained by the limited solubility of LTI-03. While it is lipophilic, and readily taken up by cells, it is hydrophobic and insoluble, which may contribute to its stability as a dry powder^15^ but make *in vitro* work challenging.

Finally, there were some limitations to using exclusively fresh IPF lung tissue biopsies. PCLS were generated exclusively from male patients (Table 2), and we were unable to process many biological tissues in parallel. Furthermore, while a longer duration of experimentation would have allowed us to better mimic a clinically relevant therapeutic dosing schedule, viability of fresh IPF PCLS tissue, cultures are time consuming and expensive, and given the induction of necrotic and apoptotic pathways by Nintedanib longer experimentation may not have been feasible. Moreover, comparison of pharmacological activity in donor PCLS, as well as an additional combination interventional therapy experimental group may have provided further insight into the mechanism and efficacy. Finally, we were limited in our analysis of epithelial-associated markers and factors, given that the IPF lung explants used to generate PCLS were largely devoid of viable type 1 and 2 epithelial cells. Studies are ongoing to explore the effect of LTI-03 on lung epithelial cell biology using IPF organoid culture systems.

**Table 2:** Key patient data and applied models and assays

Patient data				Applied models and assays			
Age	Sex	Clinical diagnosis	Pathological fibrosis pattern in explant tissue	PCLS	Col1a1 immunofluorescence	Bio-plex	Bulk RNA sequencing
53	M	IPF	UIP	•	–	•	•
58	M	IPF	UIP	•	–	•	•
60	M	IPF, PH	UIP	•	–	•	•
70	M	IPF	UIP	•	–	•	•
70	M	IPF, PH	UIP	•	•	•	–
57	M	IPF, PH	UIP	•	•	•	–
66	M	IPF	UIP	•	•	•	–
56	M	IPF	UIP	•	•	•	–

In summary, the expression profile of untreated IPF PCLS over five days in culture suggested dynamic changes in fibrotic pathways, rendering this translational model an appropriate model for testing clinically relevant antifibrotic agents. Consistent with earlier studies,^6^ this study indicates a pivotal role for cav-1 in lung fibrosis and highlights its therapeutic significance for patients with pulmonary fibrosis. This study is the first to characterize the dose-dependent effect of LTI-03 on critical canonical signaling pathways active in an IPF PCLS system. Finally, LTI-03 exhibited a similar pattern of inhibition and activation compared with Nintedanib treatment, but without the noted toxicity observed with the multi-tyrosine kinase small molecule inhibitor. At the time of this submission, LTI-03 has just completed a Phase 1b safety trial in patients with IPF (NCT05954988).

## Resource availability

### Lead contact

Further information and requests for resources and reagents should be directed to and will be fulfilled by the lead contact, BreAnne MacKenzie (breannemackenzie@reintx.com).

### Materials availability

Reagents used in this study were obtained via commercial sources listed in the STAR Methods key resources table. This study did not generate new unique reagents.

### Data and code availability

• Data reported in this article may be shared by the lead contact upon request.
• This article does not report original code.
• Additional information required to reanalyze the data reported in this article may be available from the lead contact upon request.

## Acknowledgments

We would like to acknowledge Jasmin Wagner, PhD and all of those involved in the European ILD Registry and Biobank for providing IPF patient lung tissue samples to the Guenther Lab for experimentation. We would also like to acknowledge Dale J. Christensen, PhD (Adjunct Associate Professor in the Department of Medicine, Duke University) for his insight and contributions to discussion around data generated in these studies. The research was sponsored by Rein Therapeutics, Inc.

## Author contributions

B.M., C.H., A.G., and P.M. designed the studies; P.M., C.H., and B.M. wrote the article; B.M. assembled the figures; P.M. performed PCLS and immunostaining; Y.A.P.J.S. performed all in silico, IPA, and RNA sequencing analyses; A.L.C., M.E., and B.C. assisted in the multiplex immunoassays; W.K.: provided the IPF lungs; C.H., A.G., and B.M. supervised the studies. P.M., C.H., B.M., A.G., Y.A.P.J.S., and B.C. assisted in the interpretation of the findings.

## Declaration of interests

BreAnne MacKenzie and Brian Windsor are employees of Rein Therapeutics, Inc. Dr. Cory Hogaboam and Prof. Dr. Andreas Guenther are consultants for Rein Therapeutics, Inc. Rein Therapeutics holds a patent for LTI-03 (PCT/US2021/028326), which was used in this study and developed for the treatment of IPF.

## STAR★Methods

### Key resources table

**Table:** 

REAGENT or RESOURCE	SOURCE	IDENTIFIER
**Antibodies**		

Col1a1	Rockland	Cat# 600-406-103/RRID: AB_217579

**Biological samples**		

Precision lung cut slices	European IPF Biobank or UGMLC Biobank	

**Chemicals, peptides, and recombinant proteins**		

Trizol	Thermo	15596026
RNA extraction column	Machery Nagel	740984.250
LTI-03	Lonza	Lot# AJF73B//474-33-TI344
CP	Ambiopharm	Lot# PD200120-1
Nintedanib	Boehringer Ingelheim Pharmaceuticals	#S1010

**Critical commercial assays**		

Bio-Plex Pro™ Human Inflammation Panel 1	Bio-Rad	#171AL001M

**Deposited data**		

PCLS Bulk RNA-seq	This paper	GSE305464

**Oligonucleotides**		

TapeStation	Agilent	4200/RRID:SCR_018435
Truseq stranded mRNA kit	Illumina	20020594
Illumina NovaSeq sequencer	Illumina	

**Software and algorithms**		

GenomeNet (MOTIF Search)	genome.jp	
Ingenuity Pathway Analysis	Qiagen	RRID:SCR_008653
Prism	GraphPad	V 9.0/RRID:SCR_002798
LAS-X-Core Software	Leica	v 3.7.4/RRID:SCR_013673

**Other**		

FA Fluorescent Stereoscope	Leica	M205
Bio-Plex 200 reader	Bio-Rad	RRID:SCR_018026

### Experimental methods and subject details

#### Precision cut lung slice (PCLS) cultures

Human lung tissues from idiopathic pulmonary fibrosis (IPF) patients undergoing lung transplantation and non-IPF donors were obtained via the European IPF Registry (euIPFreg) sites Vienna and Giessen and delivered via the European IPF Biobank or the UGMLC Biobank, a member of the DZL platform biobanking. The study protocol was approved by the Ethics Committee of the Justus-Liebig University School of Medicine (No. 111/08: European IPF Registry and 58/15: UGMLC Giessen Biobank), and informed consent was obtained in written form from each subject (a comprehensive list of patient data and applied assays is provided in Table 2). Lung biopsy cores were obtained exclusively from 8 patients undergoing lung transplant for treatment of IPF and processed for PCLS culture as previously described.^44^ All patients were male, an average of 61.5 years of age (std dev 6.56 years), and had a clinical diagnosis of IPF alone, or IPF with secondary pulmonary hypertension (PH). Usual interstitial pneumonia (UIP) was the main pathological finding on all explant tissue (Table 2). The models and assays performed in each PCLS culture are also summarized in Table 2. While a balance in sex is usually preferred in translational models, IPF occurs at a higher incidence rate in males than females (∼70%) therefore it was more difficult to obtain fresh transplant tissue from females with IPF.

#### PCLS treatments

LTI-03 (Sequence: FTTFTVT; Lot# AJF73B//474-33-TI344 Lonza), the inactive control peptide CP (Sequence: FAAFAVA; Lot# PD200120-1, Ambiopharm) were suspended in PCLS culture media at 0.3, 5.0, or 10 μM for treatments. Nintedanib (Boehringer Ingelheim Pharmaceuticals, Inc #S1010) was prepared in a 1 mM stock solution with sterile filtered DMSO and used at 0.1 μM or 1 μM. PCLS were treated every 12hrs and supernatant was harvested 12 h following the last of either three (day 2), seven (day 4) or nine treatments (day 5). Tissue was harvested after 9 treatments (day 5).

### Method details

#### In silico identification of caveolin-1 binding domains in human proteome

CBD motif 1 (ϕXϕXXXXϕ), CBD motif 2 (ϕXXXXϕXXϕ), and the CBD composite motif (ϕXϕXXXXϕXXϕ) were identified using the MOTIF Search tool (setting: nr-aa) available on GenomeNet (genome.jp) (1). The symbol “ϕ” represents either Tryptophan (Trp - W), Phenylalanine (Phe – F), or Tyrosine (Tyr – Y), while X is any other amino acid. These sequences were used as queries to identify protein sequences containing CBDs (2).

#### Collagen immunofluorescence quantification

Collagen 1α1 immunostaining was performed on tissue sections generated from 4 PCLS biological samples. Immunofluorescence using a col1α1 (Rockland) monoclonal antibody was performed as described.^44^ Leica M205 FA Fluorescent Stereoscope (Leica Microsystems) and LAS-X-Core Software (3.7.4. version, LAS X Life Science, Leica Microsystems) was used for imaging. PCLS were imaged under 20× magnification and 5 regions per PCLS (*n* = 4 unique biological samples) were used for analysis (almost 70% of total lung). The presence of a large bronchus or several bronchi in ROI were avoided thus limiting the size of the area available for analysis. Procedures and protocols for PCLS imaging and analysis were similar to those shown in our previous reports (Chillappagari et al.,^44^ Clin & Transl. med., 2022). Staining’s from each patient lung following respective treatments were normalized to the control PCLS of PCLS derived from the same patient. Five pictures from each stained PCLS were obtained and quantified with ImageJ on converted 16-bit images.

#### Bioplex

Proteomic analysis on 8 unique IPF PCLS supernatants collected at days 2, 4 or 5 after treatment was performed using Luminex bead-based assay kit (Bio-Plex Pro Human Inflammation Panel 1, #171AL001M, Bio Rad) according to manufacturer’s instructions. Data was acquired using a Bio-Plex 200 reader (Bio Rad) and are presented as the ratio compared to untreated (control) PCLS.

#### Bulk-RNA-sequencing

Bulk RNA sequencing was performed on tissue from 4 separate PCLS. Briefly, PCLS were minced manually in 2 mm pieces in Trizol (ThermoFisherScientific) before bead homogenization for 95 s (40 + 20+15 + 10 + 10 s) at 50 Hz. RNA was purified using a column-based workflow (Machery Nagel, 740984.250). RNA quantity and quality was assessed by RNA integrity number (RIN) via 4200 TapeStation system (Agilent). RNAseq libraries were prepared using Truseq stranded mRNA kit (Illumina; 20020594). Sequencing libraries were pooled and submitted for next-generation sequencing of 100 bp single-end reads (Illumina NovaSeq sequencer, AGCT Core; Los Angeles, CA).

#### Ingenuity pathway analysis (IPA)

Datasets containing differentially expressed genes (DEGs) were generated comparing each treatment to its respective untreated group (UT) using DESeq2 package. Files were uploaded to Ingenuity Pathway Analysis (IPA; QIAGEN Inc.) for further analysis. Venn diagrams were generated comparing a dataset of Treatment vs. UT against the respective day of Nintedanib versus UT. The top Canonical Pathways, Upstream Regulators and Disease & Function analysis were selected using the condition Nintedanib versus UT (day 2 or day 5) as reference and any features was considered significative if *Z* score was higher than 2 or lower than −2 and –log_10_(*p*-value) above 1.3. CP vs. UT and LTI-03 vs. UT data was displayed side-by-side for each analysis as comparative to Nintedanib effects over PCLS.

### Quantification and statistical analysis

All results were graphed using GraphPad Prism 9.0 Software. In Figure 2B, Tukey’s multiple comparison test was performed on mean fluorescence intensity units measured in at least 5 20X regions of 3 technical replicates of 4 biologically unique samples and error bars represent standard error of the mean.

## Supplemental information

Document S1. Figures S1–S4

Table S1. Table of human proteins containing caveolin-1 binding domains
