# Supplementary material for: LTI-03 peptide demonstrates anti-fibrotic activity in ex vivo lung slices from patients with IPF
Source: iScience. 2025 Aug 26;28(9):113437. doi: 10.1016/j.isci.2025.113437 (PMC12496166; doi:10.1016/j.isci.2025.113437)
Supplement: Document S1. Figures S1–S4 [file mmc1.pdf]

iScience, Volume *28*

Supplemental information

LTI-03 peptide demonstrates anti-fibrotic activity in *ex vivo* lung slices from patients with IPF

BreAnne MacKenzie, Poornima Mahavadi, Yago Amigo Pinho Jannini-Sa, Brecht Creyns, Ana Lucia Coelho, Milena Espindola, Clemens Ruppert, Brian Windsor, Clemens Aigner, Cory M. Hogaboam, and Andreas Guenther

Supplemental Figures and Legends

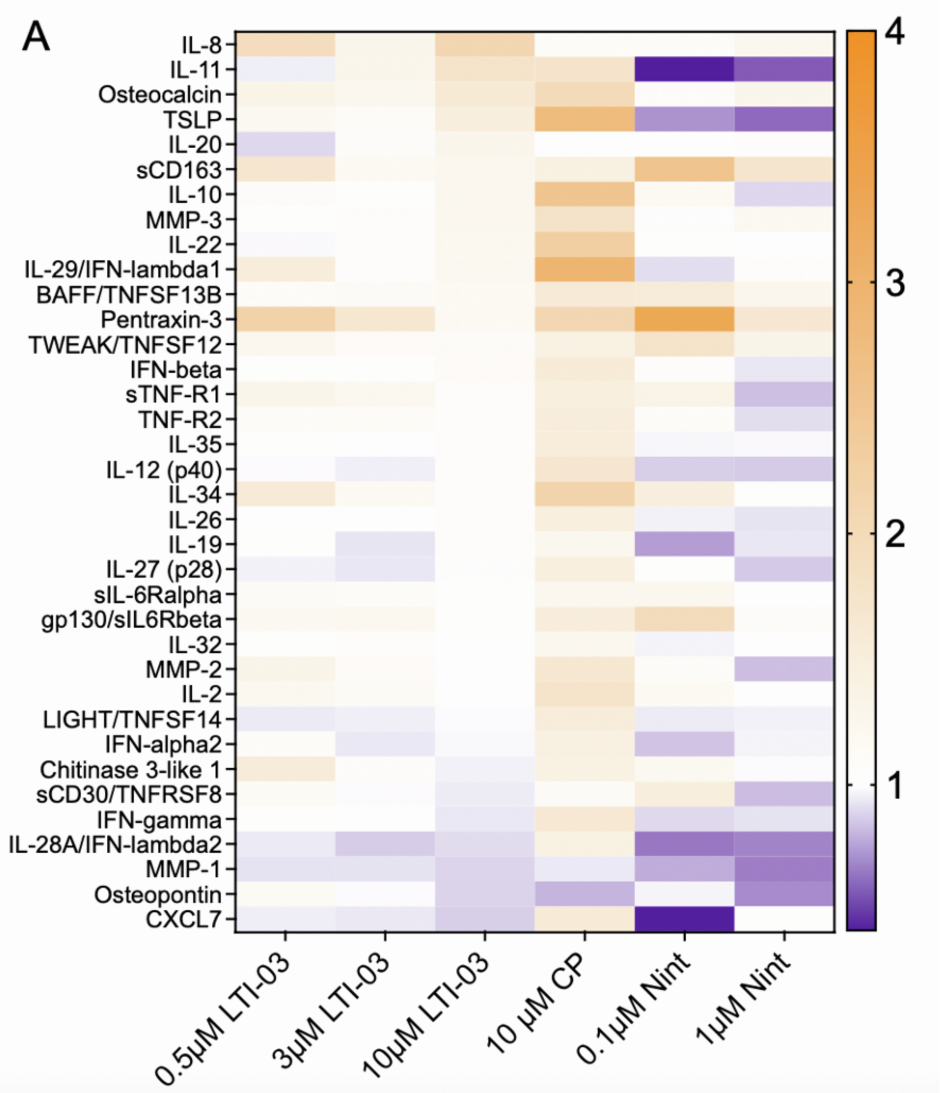

Supplementary Figure 1: LTI-03 inhibits profibrotic and inflammatory mediators in IPF PCLS following 2 days of treatment Supernatant was harvested 2 days post treatment with 0.5 μM, 3.0 μM, 10.0 μM LTI-03, 10 μM CP or 0.1 μM or 1 μM Nintedanib. (n=8/PCLS per treatment group, except 0.1 μM Nintedanib; n=4 PCLS). Heatmap indicates the mean value per protein measured and normalized to the mean of untreated controls.

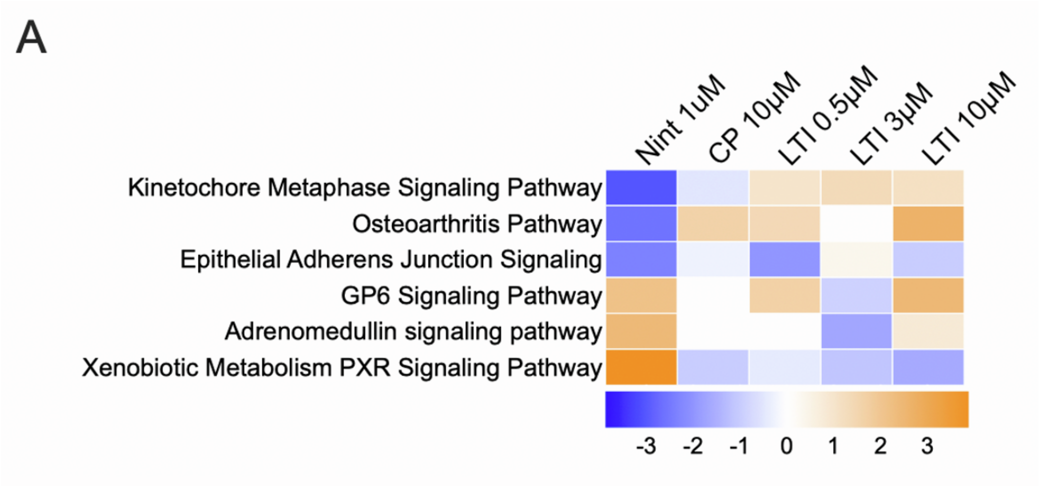

Supplementary Figure 2: Canonical pathways in IPF PCLS treated with control peptide, Nintedanib or LTI-03 after 2 days of treatment. (A) Activated canonical pathways related to fibrosis signaling in IPF PCLS tissue followed by 5 days of Nintedanib (1.0 μM), control peptide (10.0 μM) or LTI-03 (0.5 μM, 3.0 μM, 10.0 μM) treatment every 12 hours. (n=4/group). All treatments were compared to its respective untreated group and the top inactive or active canonical pathways (z-score > 2 or < -2 and log10p-val > 1.3) observed in nintedanib was used as comparison for the LTI treatments.

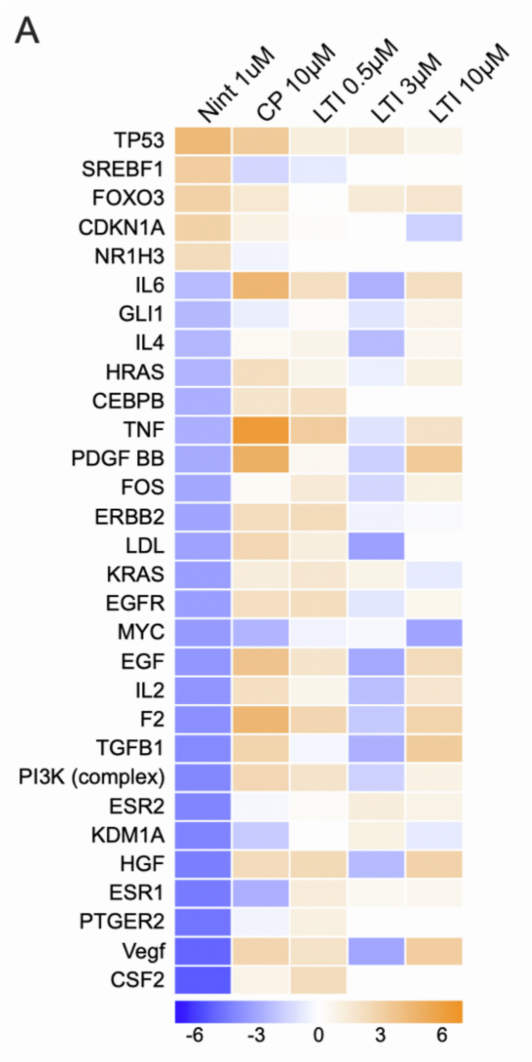

Supplementary Figure 3: Upstream regulators in IPF PCLS treated with control peptide, Nintedanib or LTI-03. (A) Expression of upstream regulators signaling related to fibrosis signaling in IPF PCLS tissue followed by 2 days of Nintedanib (1.0 μM), control peptide (10.0 μM) or LTI-03 (0.5 μM, 3.0 μM, 10.0 μM) treatment every 12 hours. (n=4/group). All treatments were compared to its respective untreated group and the top inactive or active upstream regulators (z-score > 2 or < -2 and log10p-val > 1.3) observed in nintedanib was used as comparison for the LTI-03 treatments.

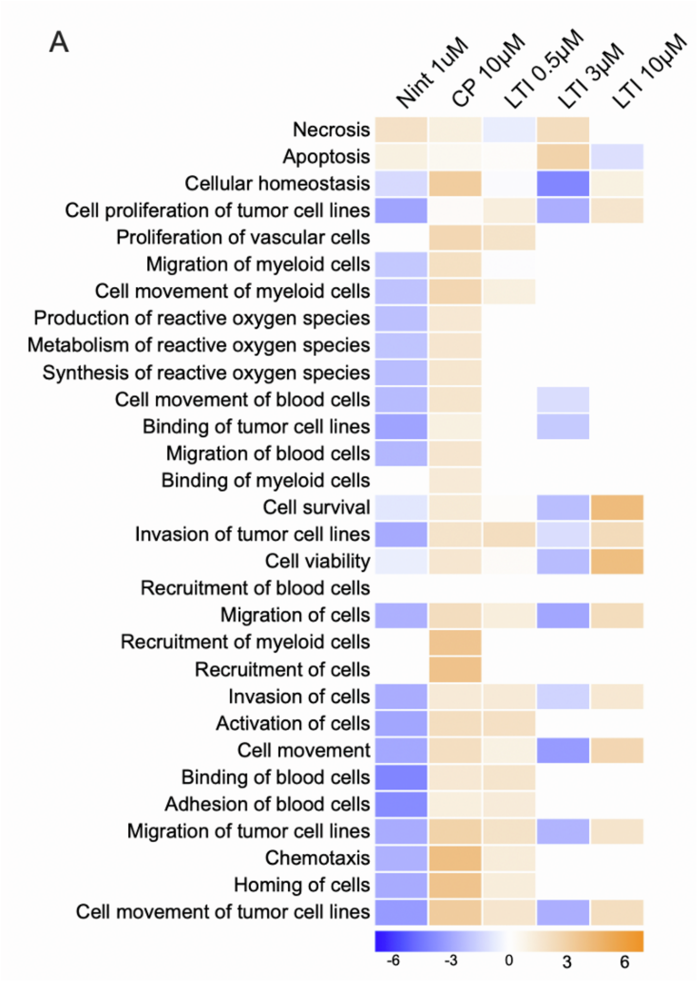

Supplementary Figure 4: Disease pathways expression in IPF PCLS treated with control peptide, Nintedanib or LTI-03. Disease pathway expression related to fibrotic signaling in IPF PCLS tissue followed by 2 days of Nintedanib (1.0 µM), control peptide (10.0 µM) or LTI-03 (0.5 µM, 3.0 µM, 10.0 µM) treatment every 12 hours. (n=4/group). All treatments were compared to its respective untreated group and the top inactive or active canonical pathways (z-score > 2 or < -2 and log10p-val > 1.3) observed in nintedanib was used as comparison for the LTI treatments.
